# Causal association between telomere length and colorectal polyps: A bidirectional two-sample Mendelian randomization study

**DOI:** 10.1097/MD.0000000000036867

**Published:** 2024-01-05

**Authors:** Yin Zhang, Jiaying Wang, Mingyu Zheng, Huanwei Qu, Shuya Yang, Fuzhou Han, Nan Yao, Wenqiang Li, Jun Qu

**Affiliations:** a Department of General Surgery, Aerospace Center Hospital, Beijing, China.

**Keywords:** colorectal polyps, Mendelian randomization, Telomere length

## Abstract

We performed a bidirectional 2-sample Mendelian randomization (MR) design to explore the causal relation between telomere length (TL) and colorectal polyps. Genome-wide association study summary data of TL and colorectal polyps were extracted from the IEU open genome-wide association study database. Single nucleotide polymorphisms were served as instrumental variables at the significance threshold of *P* < 5 × 10^−8^. The inverse variance weighted method, MR-Egger method, and weight median method were performed for causal estimation in MR. Cochran *Q* test, MR-Egger intercept test, and leave-one-out analyses were performed to evaluate the pleiotropy of the MR results. One hundred and twenty-four single nucleotide polymorphisms were selected as instrumental variables. We found significant casual association between TL and colorectal polyps. Long TL increased the risk of colorectal polyps using the inverse variance weighted method [ukb-a-521: odds ratio (OR): 1.004, 95% confidence interval (CI): 1.001–1.007, *P* = .004; ukb-d-D12: OR: 1.008, CI: 1.004–1.012, *P* < .001; finn-b-CD2_BENIGN_COLORECANI_EXALLC2: OR: 1.170, CI: 1.027–1.332, *P* = .018]. Sensitivity analyses validated that the causality between TL and colorectal polyps was robust. The study provided a causal association between TL and colorectal polyps which indicated that TL might be served as a potential biomarker of colorectal polyps for screening and prevention. Nonetheless, the conclusions need further validation.

## 1. Introduction

Colorectal polyp, a precancerous lesion, represents the most frequently encountered precursor to colorectal cancer (CRC), with approximately 70% to 90% of CRCs following the conventional “normal-polyp-cancer” progression pathway.^[1]^ The pivotal strategy for preventing the initiation of CRC is the implementation of colonoscopy screening followed by polypectomy, as it has the potential to advance the prevention window for individuals at high risk.^[2,3]^ Colorectal polyps can be categorized primarily as adenomas or serrated polyps. While the biological processes leading to the formation of adenomas and serrated polyps are not identical, they do share certain gene mutations during their development, and the transition from normal epithelial cells to either adenomas or serrated polyps typically spans several years. Enhancing comprehension of the polyp formation process facilitates the identification of risk factors associated with polyp formation, fosters the progress of CRC prevention strategies, and enables the development of more accurate preventive measures.

The telomere, a DNA-protein complex structure located at the terminus of eukaryotic chromosomes, serves the primary purpose of safeguarding chromosomes against degradation and fusion, thereby upholding chromosomal stability.^[4]^ As cellular divisions progress, telomeres undergo gradual shortening, and their length exhibits a strong correlation with the development of diverse tumors.^[5]^ Both elongated and shortened telomeres can elevate the likelihood of tumor formation. Diminished telomere length (TL) decreases chromosomal stability, increasing the likelihood of aneuploidy during cell division. However, it should be noted that cells possessing longer telomeres exhibit a greater propensity for division, consequently heightening the likelihood of genetic instability. The conventional “normal-polyp-cancer” model follows a 2-stage process involving gene mutation and chromosome instability. Presently, investigations into the correlation between TL and polyp remain within the realm of observational research, lacking a definitive conclusion.^[6,7]^

By utilizing genetic variation as an instrumental variable, Mendelian randomization (MR) effectively addresses the inherent limitations of reverse causality and confounding bias commonly encountered in observational studies.^[8]^ This study aims to employ the 2-sample MR method to investigate the causal association between TL and polyp development.

## 2. Materials and methods

### 2.1. Study design

In this study, a bidirectional 2-sample MR approach was utilized to explore the causal association between TL and the occurrence of colorectal polyps. Data related to TL and colorectal polyps were obtained from the Integrative Epidemiology Unit Open Genome-Wide Association Studies Project Database (IEU Open GWAS Database, https://gwas.mrcieu.ac.uk/).^[9]^ We set TL as exposure and colorectal polyps as outcome in the forward MR analyses. In the backward MR analyses, colorectal polyps were set as exposure and TL as outcome. The GWAS datasets for analyzing were obtained from the public platform, so no ethical approval was required.

### 2.2. The selection of instrumental variables (IVs)

Single-nucleotide polymorphisms (SNPs) were utilized as IVs. The selection of IVs should follow the 3 MR assumptions: (1) relevance assumption: there is a robust correlation between IVs and exposure; (2) exclusion restriction assumption: the IVs influence the outcome only through the exposure; (3) independence assumption: the IVs are not associated with any confounding factors. The threshold for IV selection was set at *P* < 5 × 10^−10^. Additionally, it is imperative to ascertain the absence of linkage disequilibrium among the IVs. Linkage disequilibrium between SNPs was assessed using the clumping method (*r*^2^ < 0.001, window size = 10,000 kb) on European samples sourced from the 1000 Genomes Project.

### 2.3. Statistical analysis

The inverse variance weighted (IVW) method was performed as the primary method in the MR analyses by combining Wald estimates for each SNP in a meta-analysis approach. Weighted median (WM) and MR-Egger method was performed to validate the result of IVW method.

Sensitivity analyses were performed using Cochran *Q* test, funnel plots and leave-one-out analyses. The horizontal pleiotropy was evaluated using MR-Egger intercept test. The heterogeneity was evaluated mainly by the result of Cochran *Q* test. The leave-one-out analyses were performed to evaluate the effect of each SNP in the MR results.

All analyses were performed using the 2-sample MR package (version 0.5.7) in R (version 4.3.1).

## 3. Results

### 3.1. Selection of instrumental variables

**Figure 1.**
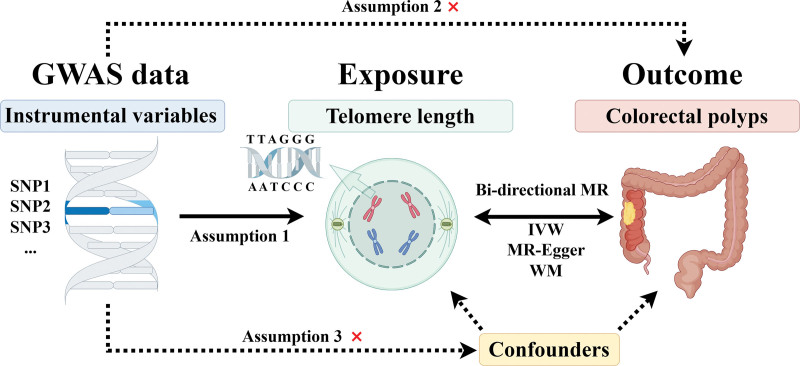
The schematic flowchart of this bi-directional 2-sample Mendelian randomization study design.

The flowchart of this study based on the 3 MR consumptions was shown in Figure 1. One GWAS dataset (id: ieu-b-4879) was selected for TL and 3 datasets (id: ukb-a-521, ukb-d-D12 and finn-b-CD2_BENIGN_COLORECANI_EXALLC) for colorectal polyps. In the dataset ieu-b-4879, the sample size was 472,174 and the number of SNPs were 20,134,421. In the dataset ukb-a-521, the sample size was 337,199 and the number of SNPs were 10,894,596. In the dataset ukb-d-D12, the sample size was 361,194 and the number of SNPs were 13,217,521. In the dataset finn-b-CD2_BENIGN_COLORECANI_EXALLC, the sample size was 184,019 and the number of SNPs were 16,380,344. Following the criteria proposed in the method section. A total of 124 SNPs was identified as IVs.

### 3.2. MR analyses of TL with risk of colorectal polyps

**Figure 2.**
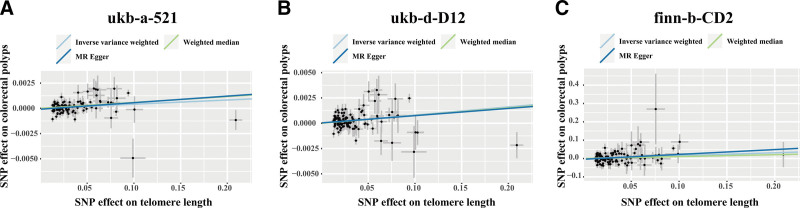
Scatter plot of Mendelian randomization based on inverse variance weighted, weighted median (WM), and MR-Egger method in the ukb-a-521 (A), ukb-d-D12 (B) and finn-b-CD2_BENIGN_COLORECANI_EXALLC (C) datasets. Scatter plots demonstrating the effect of telomere length associated SNPs on colorectal polyps on the log-odds scale. The slopes of each line represent the causal association for each method.

IVW, WM, and MR-Egger were performed to analyze TL and the risk of colorectal polyps. Using IVW method, we found TL was positively associated with the risk of colorectal polyps [ukb-a-521: odds ratio (OR): 1.004, 95% confidence interval (CI): 1.001–1.007, *P* = .004; ukb-d-D12: OR: 1.008, CI: 1.004–1.012, *P* < .001; finn-b-CD2_BENIGN_COLORECANI_EXALLC2: OR: 1.170, CI: 1.027–1.332, *P* = .018] (Table 1, Fig. 2A). The WM and MR-Egger methods both showed significant causal direction from TL to colorectal polyps which validated the robustness of the IVW results (Table 1, Fig. 2B and C). Although the result of WM method in the finn-b-CD2_BENIGN_COLORECANI_EXALLC2 dataset showed *P* > .05. The relevant trend is in accordance with other methods. The results of the forward MR analyses indicate the causal association between TL and colorectal polyps and that longer TL might increase the risk of colorectal polyps.

**Table 1:** Mendelian randomization results of telomere length on colorectal polyps.

Exposure	Outcome	Method	No. of IVs	OR (95% CI)	*P*
ieu-b-4879	ukb-a-521	IVW	109	1.004 (1.001–1.007)	.004
		MR Egger	109	1.007 (1.001–1.012)	.013
		WM	109	1.006 (1.002–1.010)	.007
	ukb-d-D12	IVW	118	1.008 (1.004–1.012)	2.191 × 10^−5^
		MR Egger	118	1.007 (1.000–1.014)	.042
		WM	118	1.008 (1.002–1.013)	.005
	finn-b-CD2	IVW	111	1.170 (1.027–1.332)	.018
		MR Egger	111	1.302 (1.026–1.651)	.042
		WM	111	1.111 (0.897–1.376)	.336

CI = confidence interval, finn-b-CD2 = finn-b-CD2_BENIGN_COLORECANI_EXALLC2, IVs = instrumental variables, IVW = inverse variance weighted, OR = odds ratio, WM = weighted median.

### 3.3. Sensitivity analyses

**Figure 3.**
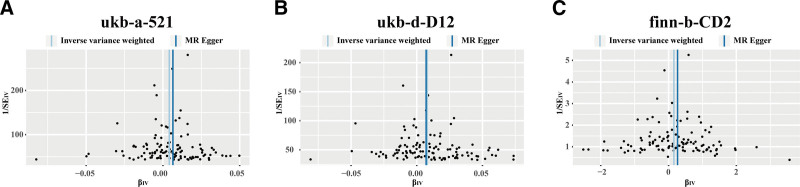
Funnel plots for telomere length on colorectal polyps using inverse variance weighted and MR-Egger method in the ukb-a-521 (A), ukb-d-D12 (B), and finn-b-CD2_BENIGN_COLORECANI_EXALLC (C) datasets.

**Figure 4.**
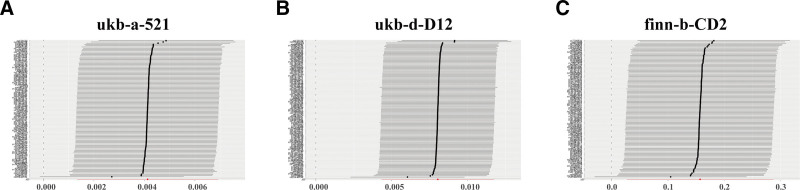
Leave-one-out analyses for telomere length on colorectal polyps in the ukb-a-521 (A), ukb-d-D12 (B), and finn-b-CD2_BENIGN_COLORECANI_EXALLC (C) datasets.

Cochran Q test was performed to evaluate the heterogeneity of IVW and MR-Egger model. Both ukb-a-521 dataset (IVW model: *P*_heterogeneity_ < 0.001; MR-Egger model: *P*_heterogeneity_ < 0.001, Table 2) and ukb-d-D12 dataset (IVW model: *P*_heterogeneity_ < 0.001; MR-Egger model: *P*_heterogeneity_ < 0.001, Table 2) showed significant heterogeneity. Finn-b-CD2_BENIGN_COLORECANI_EXALLC2 dataset showed no heterogeneity (IVW model: *P*_heterogeneity_ = 0.173; MR-Egger model: *P*_heterogeneity_ = 0.176, Table 2). Since the main IVW method was performed based on random model in this study, the heterogeneity of the 2 outcome datasets is acceptable. The funnel plots showed no significant bias in the MR analyses (Fig. 3). The leave-one-out analyses showed the combined effects of IVW model were stable (Fig. 4). None of the 3 datasets showed horizontal pleiotropy based on MR-Egger intercept test (*P*_pleiotropy_ < 0.001, Table 2). The results of sensitivity analyses showed that the causality between TL and colorectal polyps was robust.

**Table 2:** Sensitivity analyses of telomere length on colorectal polyps.

Exposure	Outcome	Method	*P*_heterogeneity_	Intercept	*P*_pleiotropy_
ieu-b-4879	ukb-a-521	IVW	4.370 × 10^−4^		
		MR Egger	4.899 × 10^−4^	8.659 × 10^−5^	.272
	ukb-d-D12	IVW	1.360 × 10^−5^		
		MR Egger	1.079 × 10^−5^	3.208 × 10^−5^	.762
	finn-b-CD2	IVW	0.172		
		MR Egger	0.175	0.004	.295

finn-b-CD2 = finn-b-CD2_BENIGN_COLORECANI_EXALLC2, IVW = inverse variance weighted, WM = weighted median.

### 3.4. Backward MR analyses

We performed backward MR analyses to avoid the reverse causality between TL and colorectal polyps. In the backward MR analyses, colorectal polyps were set as exposure and TL as outcome. 2 SNPs in the ukb-a-521 dataset, 7 SNPs in the ukb-d-D12 dataset and 3 SNPs in the finn-b-CD2_BENIGN_COLORECANI_EXALLC2 dataset were identified as IVs. Using IVW method, we found *P* > .05 in all of the 3 exposure datasets. The results of backward MR analyses showed that no reverse causality was found between TL and colorectal polyps.

## 4. Discussion

In this study, a 2-sample MR method was employed, utilizing GWAS data from the IEU database, to examine the causal relationship between TL and colorectal polyps. The forward MR analysis involved employing one dataset on TL as the exposure variable and 3 datasets on colorectal polyps as the outcome variables, revealing a significant causal association between TL and colorectal polyps. Specifically, it was observed that individuals with longer telomeres had a heightened risk of developing colorectal polyps. Sensitivity analyses were conducted, affirming the robustness and reliability of the findings. In the reverse MR analysis, with colorectal polyps as the exposure and TL as the outcome, no reverse causal relationship was found.

Adenoma, the prevailing type of colorectal polyp lesion, has been the subject of previous studies examining the correlation between TL and the occurrence of colorectal adenomas, which have produced inconclusive findings. Peacock et al conducted a study involving the measurement of TL in 40 cases of colorectal adenomas and 45 normal tissues, revealing that adenoma tissue exhibited significantly longer telomeres compared to normal tissue.^[7]^ Similarly, Hardikar and his team assessed lymphocyte TLs in 80 patients with traditional adenomas, 33 patients with serrated adenomas, 28 patients with both types of adenomas, and 49 healthy individuals (OR = 1.69, 95% CI: 1.12–2.55, *P* = .007).^[6]^ Their results suggested that short telomeres could increase the risk of serrated adenomas (OR = 2.55, 95% CI: 1.03–6.33), but there was no correlation between TL and the risk of traditional adenomas (OR = 1.58, 95% CI: 0.75–3.29).^[6]^ This implies that the influence of TL on the susceptibility to various types of colorectal adenomas may vary. Plentz and colleagues conducted an examination of the progression from adenoma to cancer and observed a significant reduction in TL during this process.^[10]^ However, it is important to note that these findings are derived from limited sample sizes and are subject to various confounding factors, resulting in inconclusive outcomes. Furthermore, no investigation into the causal relationship between TL and adenomas was undertaken. Compared to previous studies, our study employed a larger sample size, incorporated a greater number of SNPs, and employed a bidirectional 2-sample MR approach, making our findings relatively reliable.

The initial event in the development of CRC and the formation of polyp is the inactivation of the *APC* tumor suppressor gene, while subsequent chromosomal instability, characterized by mutations in *KRAS* and *TP53* genes, as well as 18q loss of heterozygosity, drives the progression from normal epithelium to polyp formation and ultimately to cancer.^[11–16]^ The primary role of telomeres is to safeguard the stability of chromosomes, whereby their shortening can directly contribute to chromosomal instability, potentially facilitating the development of CRC. Long telomeres can extend the duration prior to cellular demise, thereby augmenting the frequency of cell divisions and consequently enhancing the probability of gene mutations and chromosomal instability. However, it is important to clarify that the progression from “normal-polyp-cancer” is not an inexorable outcome nor a continuous process. While the majority of CRCs do arise from polyps, the proportion of polyps that ultimately develop into cancerous growths is only 2.6% to 5.6%. This indicates that the transition from normal epithelium to polyps and subsequently to cancer is not a seamless progression, and underscores the significance of polyps as a pivotal stage in this process.^[17]^ We previously conducted mass spectrometry experiments and proteomics analysis on the “normal-polyp-cancer” process, and the results suggest that the proteome changes in the “normal-polyp” and “polyp-cancer” processes are completely different, implying these 2 processes are relatively independent.^[18]^ It can be deduced that the findings of the study regarding the association between TL and CRC risk cannot be extrapolated to the risk of polyp occurrence. Furthermore, the development of CRC follows a temporal sequence, wherein chromosomal instability and prevalent oncogene mutations must transpire subsequent to the APC gene mutation in order to instigate CRC.^[19]^ Hence, the direct impact of short telomeres on chromosomal instability may be insufficient to induce polyp development whereas the elongation of telomeres leading to prolonged cell division times may heighten the likelihood of genetic mutations, thereby contributing to the development of polyps. This observation aligns with the findings of the present study.

Currently, the search for effective preventive measures for colorectal polyps continues to be challenging. Nonsteroidal anti-inflammatory drugs were previously regarded as effective chemical preventives, but their potential to cause gastrointestinal bleeding necessitates careful consideration, significantly restricting their widespread application.^[20–23]^ A study conducted by Hull et al utilizing a 2 × 2 multicenter, random, double-blind factorial design trial indicates that aspirin does not decrease the occurrence of polyps. However, it may exhibit a certain inhibitory impact on the recurrence of particular locations and types of polyps.^[24]^ Other preventive methods such as metformin, vitamin D, and calcium preparations are still uncertain in preventing the occurrence of colorectal polyps.^[25–29]^ The present study proposes a potential causal relationship between TL and colorectal polyps, offering a novel perspective on polyp prevention.

In addition to its role in preventing polyps, comprehending the factors contributing to polyp formation aids in the risk stratification of patients following polypectomy and routine colonoscopy screening. The duration of surveillance post-polypectomy is contingent upon the characteristics of the polyps, including size, number, location, villous architecture, and intraepithelial neoplasia grade, which are utilized to assess the likelihood of recurrence.^[30]^ It is important to note that not all patients require frequent colonoscopy surveillance. In contrast to patients with low-risk polyps, patients exhibiting high-risk factors necessitate a more expedited scheduling of colonoscopy procedures due to their heightened susceptibility to the development of metachronous colorectal neoplasia. Moreover, the precise determination of the recommended age for colonoscopy screening (typically 45 or 50 years old) requires further refinement, as the incidence rate of early onset CRC is on the rise.^[31,32]^ However, it is imperative to acknowledge that the advancement of the screening window may not possess universal applicability across all nations. In addition to individual management, it is vital to consider the economic implications of healthcare, the quantity and expertise of colonoscopists, and the extent of health insurance coverage.^[33]^ The colonoscopy screening guidelines for individuals at general risk and post-polypectomy surveillance guidelines exhibit variations among different countries. Consequently, a more effective and noninvasive approach is required to accurately determine the optimal timing for colonoscopy. In this study, the measurement of TL was conducted using leukocytes, which demonstrates its potential as a noninvasive tool for risk stratification following polypectomy and for identifying individuals who would benefit from colonoscopy screening.

It is important to acknowledge the limitations of this study. Firstly, the analysis is confined to a European population, which may restrict the applicability of the findings. Further investigation is necessary to ascertain whether these conclusions can be extrapolated to other populations. Secondly, the MR results obtained from the 3 analytical methods employed in this study suggest that the strength of the causality between TL and the development of colorectal polyps is not particularly strong. The results may be attributed to the multitude of factors that contribute to the development of colorectal polyps, potentially interacting with one another. Additionally, the inherent heterogeneity in the process of colorectal polyp development could also contribute to this observation. Nevertheless, our primary MR analyses results hold statistical significance, and both heterogeneity testing and pleiotropy testing hint at the stability of our results. The strength of this association may differ across various polyp types, necessitating additional investigation in future studies. Thirdly, it is important to note that our findings are derived from a 2-sample MR design, thus warranting the inclusion of further randomized controlled trials to confirm the causal relationship between TL and the occurrence of colorectal polyps.

## 5. Conclusion

In conclusion, a discernible correlation exists between the TL and colorectal polyps. A direct relationship is observed, whereby an elongated telomere is associated with an increased susceptibility to the development of colorectal polyps. This implies that telomeres hold promise as potential indicators for the surveillance and prevention of colorectal polyps. Future research, including more basic studies and randomized controlled trials, is needed to validate these conclusions.

## Author contributions

**Conceptualization:** Yin Zhang, Shuya Yang.

**Data curation:** Shuya Yang.

**Methodology:** Huanwei Qu.

**Software:** Mingyu Zheng, Huanwei Qu, Fuzhou Han, Nan Yao.

**Supervision:** Wenqiang Li, Qu Jun.

**Validation:** Fuzhou Han, Nan Yao.

**Visualization:** Wenqiang Li.

**Writing – original draft:** Yin Zhang.

**Writing – review & editing:** Jiaying Wang, Mingyu Zheng.
